# Moleculo Long-Read Sequencing Facilitates Assembly and Genomic Binning from Complex Soil Metagenomes

**DOI:** 10.1128/mSystems.00045-16

**Published:** 2016-06-28

**Authors:** Richard Allen White, Eric M. Bottos, Taniya Roy Chowdhury, Jeremy D. Zucker, Colin J. Brislawn, Carrie D. Nicora, Sarah J. Fansler, Kurt R. Glaesemann, Kevin Glass, Janet K. Jansson

**Affiliations:** Earth and Biological Sciences Directorate, Pacific Northwest National Laboratory, Richland, Washington, USA; Dalhousie University

**Keywords:** *de novo* assembly, Moleculo, metagenomic assembly, metagenomic binning, soil metagenomics

## Abstract

Soil metagenomics has been touted as the “grand challenge” for metagenomics, as the high microbial diversity and spatial heterogeneity of soils make them unamenable to current assembly platforms. Here, we aimed to improve soil metagenomic sequence assembly by applying the Moleculo synthetic long-read sequencing technology. In total, we obtained 267 Gbp of raw sequence data from a native prairie soil; these data included 109.7 Gbp of short-read data (~100 bp) from the Joint Genome Institute (JGI), an additional 87.7 Gbp of rapid-mode read data (~250 bp), plus 69.6 Gbp (>1.5 kbp) from Moleculo sequencing. The Moleculo data alone yielded over 5,600 reads of >10 kbp in length, and over 95% of the unassembled reads mapped to contigs of >1.5 kbp. Hybrid assembly of all data resulted in more than 10,000 contigs over 10 kbp in length. We mapped three replicate metatranscriptomes derived from the same parent soil to the Moleculo subassembly and found that 95% of the predicted genes, based on their assignments to Enzyme Commission (EC) numbers, were expressed. The Moleculo subassembly also enabled binning of >100 microbial genome bins. We obtained via direct binning the first complete genome, that of “*Candidatus* Pseudomonas sp. strain JKJ-1” from a native soil metagenome. By mapping metatranscriptome sequence reads back to the bins, we found that several bins corresponding to low-relative-abundance *Acidobacteria* were highly transcriptionally active, whereas bins corresponding to high-relative-abundance *Verrucomicrobia* were not. These results demonstrate that Moleculo sequencing provides a significant advance for resolving complex soil microbial communities.

**IMPORTANCE** Soil microorganisms carry out key processes for life on our planet, including cycling of carbon and other nutrients and supporting growth of plants. However, there is poor molecular-level understanding of their functional roles in ecosystem stability and responses to environmental perturbations. This knowledge gap is largely due to the difficulty in culturing the majority of soil microbes. Thus, use of culture-independent approaches, such as metagenomics, promises the direct assessment of the functional potential of soil microbiomes. Soil is, however, a challenge for metagenomic assembly due to its high microbial diversity and variable evenness, resulting in low coverage and uneven sampling of microbial genomes. Despite increasingly large soil metagenome data volumes (>200 Gbp), the majority of the data do not assemble. Here, we used the cutting-edge approach of synthetic long-read sequencing technology (Moleculo) to assemble soil metagenome sequence data into long contigs and used the assemblies for binning of genomes.

**Author Video**: An author video summary of this article is available.

## Abstract (precis)

Soil microorganisms carry out key processes for life on our planet, including cycling of carbon and other nutrients and supporting growth of plants. However, there is poor molecular-level understanding of their functional roles in ecosystem stability and responses to environmental perturbations. This knowledge gap is largely due to the difficulty in culturing the majority of soil microbes. Thus, use of culture-independent approaches, such as metagenomics, promises the direct assessment of the functional potential of soil microbiomes. Soil is, however, a challenge for metagenomic assembly due to its high microbial diversity and variable evenness, resulting in low coverage and uneven sampling of microbial genomes. Despite increasingly large soil metagenome data volumes (>200 Gbp), the majority of the data do not assemble. Here, we used the cutting-edge approach of synthetic long-read sequencing technology (Moleculo) to assemble soil metagenome sequence data into long contigs and used the assemblies for binning of genomes.

## INTRODUCTION

Soil metagenomics has been termed the “grand challenge,” due to the complexity and diversity of the microbial communities in these ecosystems (1–4). Soils represent one of the most diverse ecosystems on the planet (1), with estimates of billions of microbial cells and ~100,000 unique bacterial and archaeal species in 1 g of soil (2). Analysis of existing soil metagenomic data has also resulted in high microbial genetic diversity estimates, equivalent to ~10^12^ genes per gram of soil (2). This high diversity represents an enormous challenge to metagenomics, due to low coverage obtained for individual populations, uneven sampling of microbes, and the large amount of sequence data acquired, often in short DNA fragments (~100 to 150 bp) (3, 4). It has been estimated that tera-base pairs (Tbp) of sequence data would be required to adequately sample a single gram of soil if using current metagenomic sequencing and assembly platforms (3–6).

To address this challenge of soil metagenome assembly and annotation, the Department of Energy’s Joint Genome Institute (JGI) initiated the Great Prairie Soil Metagenome Grand Challenge Initiative (1), which included native prairie and adjacent long-term agricultural soil locations in Wisconsin, Iowa, and Kansas. As a result of the JGI initiative, the two largest soil metagenomes published to date are from Iowa native prairie soil, containing 3.3 billion reads, or 257 Gbp of raw data, and from the adjacent cultivated soil (“continuous corn”), containing 1.8 billion reads or 141 Gbp (4). However, only nine sequences of >10 kbp could be assembled from the continuous corn, and none was >10 kbp from the native Iowa prairie soil assembly. Approximately 80% of the sequencing data could not be assembled, and less than 11% of the reads mapped to either the assembly from the continuous corn or from the native prairie soil (4). Thus, novel genomes, genes, and functional guilds have yet to be discovered among the vast microbial diversity present in soil ecosystems.

Of the JGI soil metagenomes resulting from the Grand Challenge project, the largest amount of sequence data was obtained from the Kansas native prairie (>1.3 Tbp). Therefore, we focused on Kansas native prairie soil as the target metagenome with the aim of further advancing sequencing and assembly approaches to attempt to tackle some of the challenges inherent to soil metagenomics.

A promising approach for improving metagenome analysis is *de novo* assembly. A *de novo* assembly approach provides error correction of short reads, connects fragmented protein-coding open reading frames (ORFs), reduces data volume, and enables higher-quality functional gene annotation and better phylogenetic taxonomic assignments than do gene-centric-based approaches (4). By comparison, unassembled short reads of <150 bp offer few solutions for annotation and phylogenetic approaches, as they are often too short for reliable analysis (7), and short read length alone can cause artifacts (8). *De novo* assembly is therefore often the first step in metagenomic analysis after data trimming and cleaning (9). However, this approach to metagenomics currently has three major caveats: (i) it does not capture long-range sequence contiguity, as community DNA is fragmented commonly to <1-kbp lengths (10); (ii) it masks individual strain-level genotypes due to pooling of community DNA (10); (iii) current assembly algorithms do not scale computationally for complex soil ecosystems due to the high diversity and large data volume (4).

In addition, due to the assembly issues associated with soil, it has been difficult to reconstruct genomes from complex soil metagenomes. However, genomes have been successfully binned from lower-complexity metagenomes, including acid mine drainage (11), deep aquifers (12), mat microcosms (13), wastewater bioreactors (14, 15), permafrost soil (16, 17), and the human gut (18). Recently, the complexity of soil was reduced after incubating samples under harsh treatment conditions (e.g., in the presence of heavy metals, salts, and ethanol), and this improved metagenome assembly and allowed for direct binning of genomes (19).

A promising alternative approach to address the challenges in soil metagenome assembly is to utilize Moleculo hybrid synthetic long reads generated via the high-throughput sequencing Illumina platform, also known as Illumina TruSeq long-read hybrid subassembly (20). The Moleculo technology can provide >8-kbp subassembled contig lengths with 99.9% accuracy (20–22) and is summarized in Fig. S1A in the supplemental material. Recently, metagenomic studies conducted on sediments (23) and the human gut microbiome (24) have demonstrated that Moleculo alone, or hybrid assembly with short reads and Moleculo, can generate long contigs of >100 kbp.

10.1128/mSystems.00045-16.1 Figure S1  (A) Moleculo technology schematic and wet lab workflow (adapted from Voskoboynik et al., 2013 [18]). First, large fragments of genomic DNA (~50 to 100 kbp) are obtained, and then fragments of ~10 kbp are selected. The ~10-kbp DNA fragments are end repaired, and standard A-tailing is completed. The Illumina adapters are added to the end-repaired and A-tailed DNA fragments by ligation, which creates a long-fragment library. The long-fragment library is serially diluted to ~200 molecules per well in a 384-well plate. A long PCR is completed to clonally amplify the long DNA molecules, Illumina’s Nextera Tn*5* transposase is used to fragment and add Illumina specific adapters to the ends of the fragment molecules in each well, and then a final PCR adds bar codes per well with complete Illumina adapters for sequencing. (B) Histogram of read length distribution for the Kansas native prairie Moleculo-only library after subassembly. (C) Histogram of quality score distribution for the Kansas native prairie Moleculo-only library after subassembly was performed with FastQC (http://www.bioinformatics.babraham.ac.uk/projects/fastqc/). Download Figure S1, PDF file, 0.2 MB. Copyright © 2016 White et al. 2016 White et al. This content is distributed under the terms of the Creative Commons Attribution 4.0 International license.

The objectives of our study were (i) to evaluate whether the Moleculo technology alone would improve soil metagenome assembly and allow for genome binning from a complex soil ecosystem; (ii) to compare and contrast the Moleculo assemblies to metagenome assemblies from three different Illumina read-length formats, including short reads with 100-bp paired ends (here called short reads [SR]), rapid-mode reads of 250-bp paired ends (here called rapid mode reads [RMR]), and reads from Moleculo only, which were sequenced using rapid-mode reads of 250-bp paired ends and then subassembled; (iii) to reconstruct functionally active pathways by mapping metatranscriptome reads to the Moleculo assembly; (iv) to reconstruct functionally active genome bins by mapping metatranscriptome reads to the genome bins.

## RESULTS

### Moleculo subassembly provides long and accurate reads and improves assembly of complex metagenomes.

We analyzed a combination of existing metagenome sequence data from native prairie soil that was previously collected at the Konza Prairie Station in Kansas and sequenced at the JGI. We also performed additional sequencing of fresh soils from three additional locations of the same Kansas native prairie (sites A, B, and C). We obtained approximately 300 million raw reads (69.7 Gbp of raw data) using the Moleculo technology and the majority were long, with 15,400 contigs of >9 kbp, 5,600 contigs of >10 kbp, and a total assembly of 775 Mbp on contigs of >1.5 kbp (Table 1; see also Fig. S1B in the supplemental material). The quality of the data obtained using Moleculo was on average >Q_30_ (a Q_30_ score represents a probability of an incorrect base call of 1 in 1,000, or 99.9% accuracy), suggesting Sanger-like accuracy (see Fig. S1C).

**TABLE 1:** Assembly statistics for Moleculo-only, SR, RMR, and hybrid assembly contigs^a^

Format	Data (Gbp)	Read length (bp)	No. of contigs	Size (Mbp)	No. of reads for bin size cutoff				*N*_50_	Reads mapped (%)
					<1.5 kb	>5 kb	>9 kb	>10 kb		
Short read	109.7	100	315,254	520	196,254	4,683	827	604	1,593	31.3
Rapid mode read	87.7	250	743,563	1,200	462,165	8,532	936	584	1,577	61.3
Moleculo only	69.6	250	123,695	775	0	82,777	15,479	5,647	7,781	98.6
Hybrid	0.82	(>4-kb contigs)	109,623	820	0	90,879	20,325	10,198	7,915	38.1

a “Moleculo only” indicates the subassembled contigs from the TruSeq synthetic long-read DNA library prep (Illumina). Values in the column for data size indicate the total raw data obtained with each format. Read lengths for short reads are in the HiSeq 100-bp paired-end format, whereas rapid mode reads and Moleculo-only reads were sequenced in the HiSeq rapid mode in the 250-bp paired-end format. Size represents the total assembly size, and “Reads mapped” data represent the total unassembled reads mapped to contigs of >1 kbp in length overall, determined using Bowtie2. The number of contigs of >5 kb includes all contigs for the given length cutoff.

We also compared the Moleculo data to metagenome assemblies obtained using SR 100-bp paired-end data, previously generated at the JGI, and Illumina HiSeq RMR 250-bp paired-end reads (Table 1). The MegaHit assembler was used to assemble the data from the SR and RMR sequencing approaches, resulting in >30% overall read mappability to contigs of >1 kbp in length (Table 1) (25). The SR assembly resulted in 827 contigs of >9 kbp, 604 contigs of >10 kbp, and a total assembly of 520 Mbp on contigs of >1 kbp. The RMR assembly resulted in 936 contigs of >9 kbp, 584 contigs of >10 kbp, and a total assembly of 1.2 Gbp on contigs of >1 kbp. Moleculo had 98.6% overall read mappability or alignment to contigs of >1.5 kbp in length (as none was below 1.5 kbp), suggesting most of the read data were represented in contigs (Table 1). Among Moleculo-only contigs, 67% were >5 kbp; by comparison, <1.5% of either the RMR or SR assemblies were represented in contigs of >5 kbp (Table 1). In addition, Moleculo alone provided ~10 times more long contigs (>10 kbp) than either SR or RMR assemblies (Table 1).

A hybrid assembly method was used to combine de Bruijn graph (i.e., MEGAHIT) (25), string graph (i.e., Illumina’s TruSeq long-read assembly app, v. 1.0), overlap consensus (i.e., CAP3) (26), and minimus2 (27, 28) to merge assembly contigs. SR and RMR were assembled with MEGAHIT, whereas Moleculo was assembled with the TruSeq long-read assembler. We pooled >4-kbp contigs from SR, RMR, and Moleculo, assembled them with CAP3, and completed a final contig merge with the minimus2 program. The resulting hybrid assembly had >10,000 contigs that were >10 kbp (Table 1).

### Full-length rRNA gene sequences captured by Moleculo in native prairie soil metagenomes.

**FIG 1:**
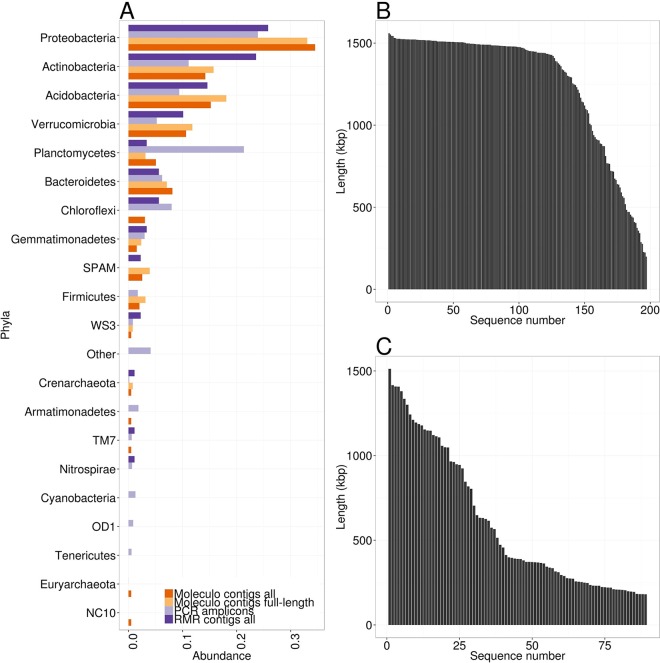
16S rRNA gene abundances and length distributions across various sequencing technologies. (A) 16S rRNA gene abundances across Moleculo-only and RMR contigs or from PCR amplicon sequencing (I-Tags). 16S rRNA genes from RMR contigs include all sequences, including the few full-length sequences of >1.4 kbp. 16S rRNA genes from Moleculo-only contigs include all that were <1.4 kbp and those that were full-length 16S rRNA sequences (>1.4 kbp). (B) 16S rRNA gene length distribution from Moleculo-only contigs. (C) 16S rRNA gene length distribution from RMR contigs.

The 16S sequences obtained from the Moleculo sequence assembly included representatives of the main bacterial taxonomic branches in the Kansas native prairie ecosystem, based on comparison to 16S rRNA PCR amplicon data. The majority (>95%) of the microbial diversity in the amplicon data was represented by 16 phyla and candidate phyla: *Acidobacteria*, *Actinobacteria*, *Armatimonadetes* (formerly OP10), *Bacteroidetes*, *Chloroflexi*, *Cyanobacteria*, *Firmicutes*, *Gemmatimonadetes*, OD1 (candidate), *Nitrospirae*, TM7 (candidate), *Planctomycetes*, *Proteobacteria*, *Tenericutes*, *Verrucomicrobia*, and WS3 (candidate) (Fig. 1A), and we reconstructed full-length 16S rRNA gene sequences from 11 of these phyla (Fig. 1A). *Armatimonadetes*, *Cyanobacteria*, *Tenericutes*, OD1, and TM7 phyla were also detected by Moleculo but were not full length (Fig. 1A). Interestingly, both the RMR and Moleculo sequencing technologies captured the candidate SPAM (i.e., spring alpine meadow) phylum, whereas the amplicon data did not (Fig. 1A). In contrast, the amplicon data predicted a much higher *Planctomycetes* abundance than was represented in the Moleculo contigs (Fig. 1A), suggesting possible PCR bias in the primers for these groups. The Moleculo data also contained full-length 16S rRNA gene sequences from both abundant members (e.g., *Acidobacteria*, *Actinobacteria*, *Proteobacteria*, *Verrucomicrobia*) as well as rare members of the community (e.g., WS3, *Crenarchaeota*, *Nitrospirae*) (Fig. 1A).

It has been challenging to reconstruct full-length 16S rRNA gene sequences from complex metagenomes, with few successful examples to date (29). Here, we compared the RMR sequence assembly to the Moleculo subassembly for achievability of longer 16S rRNA gene sequences. The Moleculo assembly yielded double the number of positive 16S rRNA gene identifications (197) (Fig. 1B) than the RMR assembly, with an average sequence length of ~1,268 bp; 127 were full-length sequences of ~1,400 bp or greater (Fig. 1B). In contrast, RMR assembly captured only 89 assembled 16S rRNA sequences, with an average sequence length of ~597 bp, and only five sequences were obtained that were >1,400 bp (Fig. 1C).

### Moleculo subassembly captures of enzyme functional potential in a complex soil community.

The Moleculo metagenome annotation was completed by using Metapathways2 to generate a MetaCyc pathway genome database (ePDGB) with pathway tools for Kansas native prairie soil (30). Of the 354,863 genes in the Moleculo metagenome annotation, 112,704 (32%) were assigned an Enzyme Commission (EC) number, and of the 5,684 official EC enzymes, 1,461 (26%) were assigned to at least 1 gene in the Moleculo metagenome annotation. Of the 2,260 MetaCyc pathways identified from 2,600 organisms, 352 (16%) were predicted for the Moleculo metabolic annotation.

**FIG 2:**
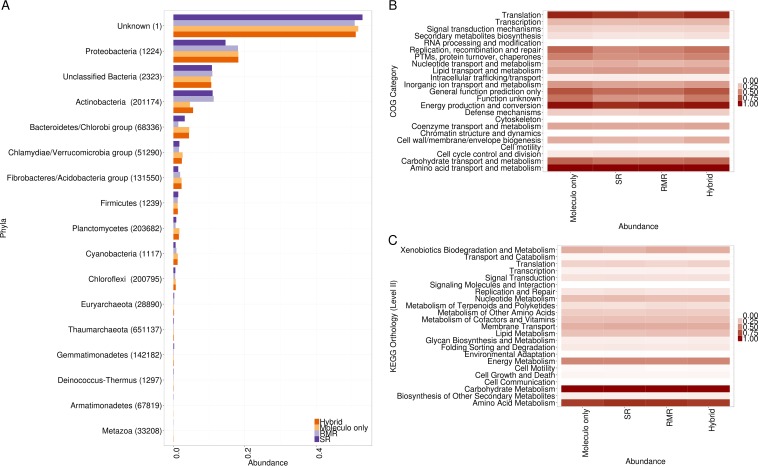
The predicted functional and taxonomic annotations and assignments for the four various assembly formats, including SR, RMR, Moleculo-only, and hybrid assembly contigs. (A) Bar graph of RefSeq taxonomic annotations. (B) Heat map of COG functional annotations. (C) Heat map of KEGG orthology (KO) functional annotations.

We annotated the functional potentials for enzymes in each of the four assemblies (SR, RMR, Moleculo only, and hybrid) by using the Kyoto Encyclopedia of Genes and Genomes (KEGG; http://www.genome.jp/kegg/) and Clusters of Orthologous Groups (COG; http://www.ncbi.nlm.nih.gov/COG/) ontology assignments, and we obtained taxonomic assignments using RefSeq (http://www.ncbi.nlm.nih.gov/refseq/). We found that both the taxonomic and functional potentials among various assemblies were quite similar (Fig. 2A to C), although there were slightly more *Actinobacteria* in the RMR and SR assemblies than in the Moleculo-only and hybrid assemblies (Fig. 2A).

**FIG 3:**
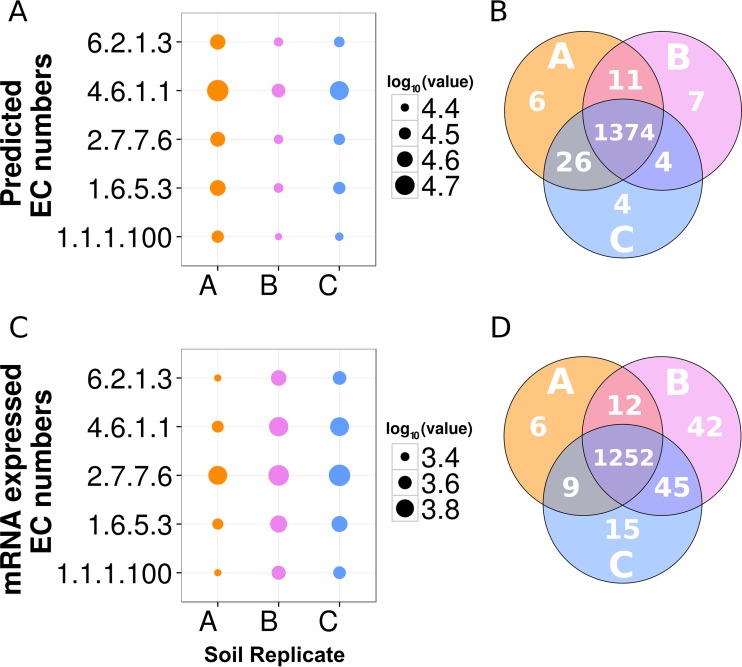
MetaCyc EC annotation abundances for Kansas native prairie soil samples A, B, and C for metagenomes and metatranscriptomes mapped to Moleculo assembly. EC numbers and activities: 1.1.1.100, 3-oxoacyl-(acyl-carrier-protein) reductase; 1.6.5.3, NADH:ubiquinone reductase H^+^-translocating; 2.7.7.6, DNA-dependent RNA polymerase; 4.6.1.1, adenylate cyclase; 6.2.1.3, long-chain fatty acid-CoA ligase. (A) The top five predicted EC number counts. (B) Venn diagram of the predicted EC counts. (C) The top five expressed EC number counts in the metatranscriptome data. (D) Venn diagram of the EC number counts from the mapped metatranscriptomes.

Three separate metagenomes were sequenced using RMR from three different sampling locations in the Kansas native prairie (soil locations A, B, and C). The RMR sequence reads from the different sampling locations were then mapped back to the Moleculo contigs by using Bowtie2 (31); of those, 23.04% of location A, 21.34% of B, and 24.48% of C aligned to the Moleculo contigs. In all three soil locations, the top five most abundant enzyme activities (EC numbers) predicted from mapping to the Moleculo assembly were long-chain fatty acid coenzyme A (CoA) ligase activity (EC 6.2.1.3), adenylate cyclase activity (EC 4.6.1.1), DNA-dependent RNA polymerase (EC 2.7.7.6), NADH dehydrogenase (ubiquinone) activity (EC 1.6.5.3), and 3-oxoacyl-(acyl-carrier-protein) reductase (NADPH) activity (EC 1.1.1.100) (Fig. 3A). Of the 1,461 unique ECs that mapped to the Moleculo contigs, 1,374 (94%) were shared by soil metagenomes A, B, and C, with less than 1% being unique to just one of the A, B, and C metagenomes (Fig. 3B).

Metagenome data only reveal information about the metabolic potential of a system, but not all genes are expressed at any given time. Therefore, we sequenced RNA (i.e., metatranscriptomes) from Kansas soils A, B, and C by using the RMR format and found that 26 to 27% of the metatranscriptome sequencing (whole-community RNA-Seq) reads from each of the soil replicates could be mapped to the Moleculo assembly, suggesting that a quarter of the genes were expressed at the time of sampling. Of the 1,461 EC numbers that were annotated in the Moleculo-only assembly, 1,391 (95%) were expressed in at least one soil sample and 1,252 (86%) were expressed in all three samples (Fig. 3D). Less than 2% of the expressed proteins with EC assignments were unique to any one of the three samples (Fig. 3D).

### Moleculo subassembly provides genomic resolution via binning.

We compared binning quality by using the resulting assemblies from the three assembly methods: SR, RMR, and Moleculo only. We obtained 129 genomic bins from the Moleculo-only data, based on matching 107 hidden Markov model (HMM) marker genes that cover ~95% of all bacteria, with an average marker count of 1.39 for each marker per bin using the MaxBin binning system (see Fig. S2 in the supplemental material) (32–34). The average completeness (the percentage of 107 HMM marker genes covered) was ~2% higher with Moleculo than with SR assemblies and ~16% higher than with RMR assemblies (Table 2). The RMR assembly resulted in more genome bins (225 bins) than either the Moleculo or SR assemblies, with 129 and 110 bins, respectively, for the 107 HMM genes (Table 2). Of the three assemblies, Moleculo had generally higher average genome completeness on fewer longer contigs.

10.1128/mSystems.00045-16.2 Figure S2  Histograms using the binning statistics from MaxBin marker gene abundances using 107 hidden Markov models (HMMs) for the Moleculo-only subassembled contigs. Plots were prepared to summarize the unique markers (A), total markers (B), and average marker count for each marker per bin (C). Download Figure S2, PDF file, 2.8 MB. Copyright © 2016 White et al. 2016 White et al. This content is distributed under the terms of the Creative Commons Attribution 4.0 International license.

**TABLE 2:** Metagenomic binning statistics and obtained bins for the various assembly formats^a^

Method	No. of bins	Avg % abundance	Avg % completeness	Avg genome size (Mbp)	% G+C
Short read	110	37.91	38.69	4.03	63.44
Rapid mode	225	2.58	24.26	2.73	65.29
Moleculo only	129	82.53	40.81	4.65	57.82

a Moleculo only, short read, and rapid mode reads were obtained using MaxBin (32, 33). Abundance, percent completeness, genome size, and G+C content data are average results from the various assembly formats, calculated from the MaxBin resulting output statistics. Abundance was calculated as the average read map coverage across a bin. Completeness was calculated as a percentage of the number of unique hidden Markov marker genes (107 markers used here). For example, if a bin had 53 unique marker genes detected, that means roughly 50% genomic completeness, based on the 107 marker genes that cover 95% of all bacteria (34). Genome size is predicted by the grouped contigs per bin, based on the *N*_50_ value and bin genome assembly size. G+C content was calculated per bin by using MaxBin (32, 33).

**FIG 4:**
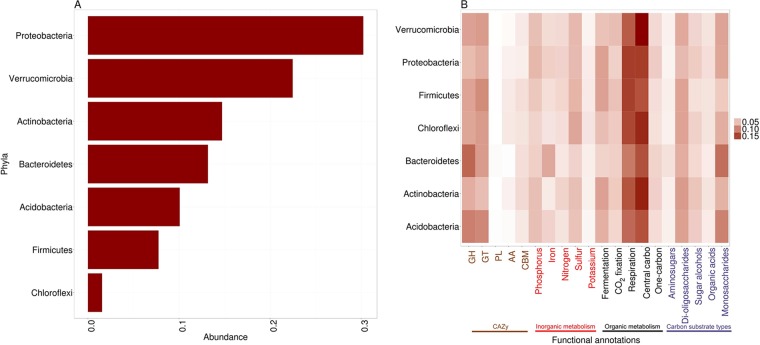
Taxonomic and phylogenetic classifications of genome bins and their metabolic potential. (A) Taxonomic and phylogenic classifications of genome bins, determined using JspeciesWS. (B) Metabolic potential of the bins. Data were normalized for intraphylum protein-coding gene counts. CAZY family abbreviations: CBM, carbohydrate-binding module; AA, auxiliary activities; PL, polysaccharide lyases; GT, glycosyltransferases; GH, glycoside hydrolase.

Binning via use of MaxBin captured representative genome bins of all of the dominant phyla that were predicted from the 16S rRNA sequences obtained using the Moleculo assemblies. Phylogenetic identifications of 129 genomic bins obtained from Moleculo-only data included *Proteobacteria* (39 bins, or ~30%), *Verrucomicrobia* (29 bins, or ~22%), *Actinobacteria* (19 bins, or ~15%), *Bacteroidetes* (17 bins, or ~13%), *Acidobacteria* (13 bins, or ~10%), *Firmicutes* (10 bins, or ~8%), and *Chloroflexi* (2 bins, or ~1.5%) (Fig. 4A). All of the bins represented novel, previously undescribed bacteria, based on their sequence similarities to isolate genomes in databases (see Table S1 in the supplemental material). Among the proteobacterial bins, ~59% were most similar to representatives of nitrogen-fixing *Alphaproteobacteria* (i.e., *Bradyrhizobium* and *Mesorhizobium*). The 29 *Verrucomicrobia* bins were most closely related to a reference genome in the *Spartobacteria* class (see Table S1). The *Actinobacteria* bins were classified as *Frankia*, *Solirubobacter*, *Nocardioides*, and *Geodematophilaceae* (see Table S1). All the *Acidobacteria* bins had closest similarity to a reference genome candidate, uncultured *Acidobacteriaceae* bacterium KBS83 (see Table S1).

10.1128/mSystems.00045-16.6 Table S1  Bins with phylogenetic identification based on JspeciesWS tetra correlation search (TCS). Download Table S1, XLSX file, 0.01 MB. Copyright © 2016 White et al. 2016 White et al. This content is distributed under the terms of the Creative Commons Attribution 4.0 International license.

The genome bins contained a diversity of metabolic potential for metabolism of complex macromolecules and elemental cycling. Functional annotations of the bins suggested that all of them had diverse glucoside hydrolyases (GHs) and glycosyltransferases (GTs), predicted by CAZy notation (Fig. 4B). All bins also had high abundances of predicted protein-coding ORFs for cycling of carbon through central carbohydrate metabolism and cellular respiration (Fig. 4B). The *Acidobacteria* and *Bacteroidetes* bins had higher abundances of GHs and genes related to monosaccharide utilization (Fig. 4B). The *Bacteroidetes* were also predicted to have more iron-related pathways than other bins (Fig. 4B), mainly from siderophores and transporters.

We obtained the first complete genome from a native complex soil by using metagenome binning. The genome bin is taxonomically related to the genus *Pseudomonas* (see Fig. S3 in the supplemental material), specifically, “*Candidatus* Pseudomonas sp. strain JKJ-1.” Based on its full-length 16S rRNA gene sequence (1,531 bp), its closest relatives in the NCBI database include *Pseudomonas fluorescens* strain FW300-N2E3 (accession number CP012830; 89% identity). “*Candidatus* Pseudomonas sp. strain JKJ-1” is a complete genome of 6,408,606 bp, with 6,128 protein-coding open reading frames (see Fig. S4 in the supplemental material), 59 tRNAs, and 1 rRNA operon, based on RAST annotation (35).

10.1128/mSystems.00045-16.3 Figure S3  The KBase species tree for “*Candidatus* Pseudomonas sp. strain JKJ-1,” based on RAST annotation predictions and determination of conserved COG (Clusters of Orthologous Groups) domains (http://www.ncbi.nlm.nih.gov/COG/), plotted using FastTree2 (45). “*Candidatus* Pseudomonas sp. strain JKJ-1” was compared to 200 *Pseudomonas* species. Download Figure S3, PDF file, 0.6 MB. Copyright © 2016 White et al. 2016 White et al. This content is distributed under the terms of the Creative Commons Attribution 4.0 International license.

10.1128/mSystems.00045-16.4 Figure S4  Circular genome map of “*Candidatus* Pseudomonas sp. strain JKJ-1.” CDS is the coding DNA sequence (a protein-coding open reading frame) that is equivalent in prokaryotes due to lack of introns. CDS are the outermost two blue rings. The next ring in black is the G+C content. The green and purple rings are GC content skewed positive (green) and negative (purple) in a strand of DNA. The innermost and outermost rings are marked with bold tick marks, incidating 1 Mbp. Download Figure S4, PDF file, 0.6 MB. Copyright © 2016 White et al. 2016 White et al. This content is distributed under the terms of the Creative Commons Attribution 4.0 International license.

### Genome bins provided by Moleculo subassembly captured metabolically active members of the community.

**FIG 5:**
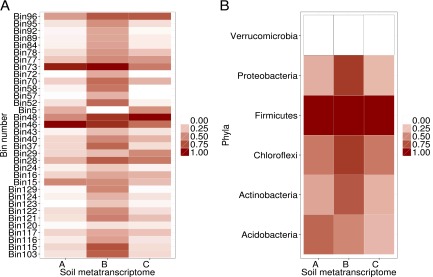
Heat map of mRNA reads mapped to genome bins and phylum-level bin averages of transcriptional activity. (A) mRNA bin read abundances across the most highly transcriptionally active bins. (B) Grouped bins by phylum-level mRNA expression abundances, rescaled to abundances based on whole mRNA counts per metatranscriptome per bin, or averaged from all the bins in a phylum group.

Metatranscriptome mRNA reads were mapped to the genome bins as a means of determining which of the represented microbes were transcriptionally active (Fig. 5). Some of the genome bins had many transcript reads that could be mapped, whereas others contained very few. Genome bins from soil sample B were more transcriptionally active than those from samples A or C (Fig. 5). Bins 46 and 73, both *Acidobacteria* bins, were highly transcriptionally active across the samples (Fig. 5A). Although *Verrucomicrobia* were highly abundant based on the 16S rRNA analysis (Fig. 1) and bin abundance (Fig. 4A), few transcripts mapped back to the *Verrucomicrobia* bins (Fig. 5B), suggesting that the represented microbes were dormant or otherwise in a state of low transcriptional activity at the time of sampling.

## DISCUSSION

Here, we successfully applied the Moleculo technology to address one of the biggest challenges facing metagenomics: the assembly of highly complex soil microbial communities (36). Using Moleculo sequencing alone, we obtained ~70 Gbp of data with more than 5,000 reads longer than 10 kbp, and >95% of the reads could be mapped back to subassembled data (~1.5 kbp in length). By comparison, using SR sequencing, assembly of the Iowa prairie soil, with >400 Gbp of data, provided only 9 contigs that were >10 kbp, with only 10.72% of the reads mapping to contigs (4). Here, we found that via hybrid assembly of all of the existing sequence data from Moleculo, SR, and RMR technologies (~267 Gbp), more than 10,000 contigs were obtained that were >10 kbp in length. Thus, this represents the highest-quality and most extensive metagenome assembly of a soil microbial community to date.

Moleculo library preparation is currently more expensive (~10× more) than use of standard shotgun libraries. However, a single library sequenced using the Moleculo technology represents a reduction in the amount of sequencing throughput required to obtain longer contigs, with ~9× more >10-kbp contigs than with SR or RMR sequencing technologies. The hybrid assembly nearly doubles the number of long contigs that are >10 kbp compared to Moleculo alone and increases by ~17× the number compared to SR or RMR alone. Moleculo also provides a cost savings by reducing downstream assembly costs for complex metagenomes. Another benefit of Moleculo is lower DNA input requirements (~500 ng) and DNA molecule lengths of ~10 kbp, which is the average length obtained using standard protocols by the Earth Microbiome Project (EMP) (http://www.earthmicrobiome.org/). Currently, we recommend that for large studies with many samples, it would be appropriate to use standard metagenomic sequencing pipelines and then to add a smaller number of Moleculo libraries to facilitate assembly only for highly complex ecosystems in order to save costs. However, if cost is not an issue, incorporation of at least some Moleculo data will help improve assembly and facilitate binning for basically any environmental sample metagenome.

Chimeras and misassembled contigs are possible from metagenomic and/or metatranscriptomic assemblies. Although the Moleculo technology results in “excellent metagenomic assemblies” (37), the choice of assembler used for subassembling Moleculo reads matters more than the diversity or complexity of the sample when it comes to quality and potential for misassemblies (37, 38). Although there are few publications to date describing use of the Moleculo technology, those that have been reported have found no evidence of chimeras (23) and few misassemblies in Moleculo contigs (23, 24). Sharon et al. (23) found no evidence of chimeras when mapping Moleculo subassembled contigs to a short-read-generated genomic bin and only two sequences were misassembled. Kuleshov et al. (24) also found low misassemblies from Moleculo technology, as “accuracy was high, with less than 0.5% of reads misassembled.” Kuleshov et al. (24) suggested that combining two complementary sequencing technologies, such as SR and/or RMR with Moleculo data, could enhance the overall quality of the assemblies, and this is supported by our findings.

This is the first successful demonstration of binning of hundreds of genomes from a highly complex soil type. We predict that even more complete genomes would have been obtained from lower-complexity samples or by adding additional Moleculo data for high-complexity samples. Assembly of the highly accurate long contigs from Moleculo resulted in 129 genome bins with a low marker gene average for each marker per bin. Many diverse phyla were represented in the genome bins, including a complete genome for a novel pseudomonad (“*Candidatus* Pseudomonas sp. strain JKJ-1”). The majority of bins were related to common soil microbes, including nitrogen-fixing *Proteobacteria* (i.e., *Rhizobium*), but many of the genomes represent phyla with few cultured representatives, including *Verrucomicrobia* and *Acidobacteria*. Of the *Verrucomicrobia* bins, the closest reference genome was from the *Spartobacteria* class, which represents a globally dominant soil group that is poorly understood (39). The *Acidobacteria* bins were classified to the closest known representative reference genome as a candidate uncultured *Acidobacteriaceae* bacterium KBS83, reflecting the paucity and likely microdiversity of genomes from this phylum. It has been suggested that *Acidobacteria* on average constitute 20% of all the bacteria in soil (40); however, with few cultured representatives, metagenome binning or single-cell genomics may be the best route to obtain genomes from within this phyla.

The majority of the metabolic predictions in the genome bins suggest that the carbon utilization pathways are very similar across the genomes. This is likely due to the diverse carbon profiles found in prairie soils that have originated from plant root exudates, decaying plant matter, or other organic material (41). These plant root exudates or other rhizosphere inputs likely shape the microbial composition and functional pathways carried out by the native prairie soil community (41).

We mapped metatranscriptome data to predict which of the genome bins represented active members of the soil ecosystem. Genome bins from *Firmicutes*, *Proteobacteria*, and *Actinobacteria* were the most transcriptionally active based on the high number of RNA-Seq reads that mapped to them. Interestingly, although *Acidobacteria* and *Verrucomicrobia* both had high relative abundances (on the DNA level) in the soils, the *Verrucomicrobia* bins showed little transcriptional activity (negligible mapping of RNA-Seq reads) whereas the *Acidobacteria* bins showed high transcriptional activity. Recent data, using propidium monoazide (PMA)-treated soil, suggest that on average 40% of the prokaryotic and fungal DNA in soil is relic DNA representing microbes that had previously lived in the soil but are not metabolically active (42). Upon PMA treatment and once relic DNA was removed, the number of *Verrucomicrobia* operational taxonomic units (OTUs) decreased (42). These data support our findings and suggest that the *Verrucomicrobia* represented by the genomic bins were dormant or relic DNA that had low activity at the time of sampling, in contrast to the *Acidobacteria*, which were highly transcriptionally active at the time of sampling.

Recovery of full-length 16S rRNA gene sequences from metagenomes has been regarded as an “outstanding challenge” (43), due to short error-prone reads and repetitive regions of rRNA that are troublesome for *de novo* assemblers. Here, by use of long-read Moleculo sequencing technology, we were able to reconstruct more than 100 full-length (~1.4-kbp) 16S rRNA gene sequences from complex soil metagenomes for the first time. The previous best example was from an oil reservoir metagenome, an environment with lower microbial diversity than soil, where 57 unique 16S rRNA gene sequences of approximately 960 bp in length were recovered (44). We noticed a higher abundance of *Planctomyces* in our 16S PCR amplicon data than in our metagenome assemblies. Our hypothesis is that because *Planctomycetes* and *Verrucomicrobia* are in the same PVC superphylum (*Planctomycetes-Verrucomicrobia-Chlamydiae*), the primers could amplify members that are hard to resolve in short paired-end reads (~250 bp) but that are possible to resolve in the longer sequences obtained in the metagenome assemblies. In addition, the candidate SPAM phylum evaded detection in our 16S amplicon analysis due to poor forward primer matches in those sequences, but it was detectable in the Moleculo assemblies. Moleculo is thus able to recover many 16S rRNA genes that are nearly or fully complete, directly from complex microbial samples such as soil, with less bias than amplicon-based sequencing.

Finally, Moleculo subassembly and/or hybrid assembly should provide a better scaffold for other database-dependent multi-omics data sets (e.g., metaproteomics and metatranscriptomics). The high-resolution database curated from Moleculo reads allows for near-genome-level annotation from a highly complex microbial community located in the Kansas native prairie and binning of hundreds of microbial genomes from a native prairie soil.

Given these results from a highly complex soil community, we anticipate and encourage the application of Moleculo technology as a broadly applicable approach for resolving highly complex microbial ecosystems across a range of sample types, to the genome scale.

## MATERIALS AND METHODS

### Site description and sample collection.

Soil samples were collected from the upper 20 cm of soil at three watershed locations at the Konza Prairie Biological Station (KPBS), a long-term ecological research (LTER) site located in eastern Kansas, USA. Each soil collected represented a field replicate, here referred to as samples A, B, and C, and was shipped on ice to PNNL, where samples were immediately sieved (2 mm^2^) to remove root fragments, aliquoted into 50-g portions in Falcon tubes, immediately frozen under liquid nitrogen, and stored at −80°C until use.

### Nucleic acid extraction (DNA and RNA).

DNA and RNA were extracted using the PowerSoil DNA and RNA isolation kits, respectively, from MoBio (Carlsbad, CA, USA) in accordance with the manufacturer’s instructions. RNA was DNase treated using Turbo DNase (Life Technologies, Grand Island, NY, USA), and then samples were purified by phenol-chloroform extraction followed by ethanol precipitation. Samples were quantified using the Qubit Fuorometer 2.0 (Invitrogen, Carlsbad, CA, USA), quality checked using a NanoDrop apparatus (Thermo Fisher, Waltham, MA, USA) and Bioanalyzer Pico RNA and HighSens DNA chips (Agilent, Santa Clara, CA, USA).

### 16S rRNA I-Tag sequencing.

Amplification of the V4 region of the 16S rRNA gene was performed in accordance with the Earth Microbiome Project recommendations (46–49), using primers 515f/926r, and sequencing was performed on an MiSeq sequencer (Illumina, San Diego, CA, USA). The QIIME pipeline (version 1.9.1) was used to demultiplex and quality filter data after sequencing. The VSEARCH (version 1.9.10) implementation of UCHIME *de novo* was used for chimera removal, followed by *de novo* OTU clustering (45–48). Of the total unique amplicons, ~22% were flagged as chimeric and were removed from any further analysis. Taxonomy annotation and tree building were completed with QIIME defaults, which make use of the Greengenes database (http://greengenes.lbl.gov), UCLUST (49), and FastTree2 (50).

### Metagenome and metatranscriptome library preparation.

Replicate metagenomes and metatranscriptomes were constructed in triplicate for Kansas soils A, B, and C. DNase-treated and purified RNA was reverse transcribed using the SuperScript VILO cDNA synthesis kit (Life Technologies, Grand Island, NY, USA), according to the manufacturer’s protocol, using a 1:1 mixture of 1 µM random hexamers/decamers. Second-strand synthesis was performed using the NEBNext mRNA second-strand synthesis module (NEB, Ipswich, MA, USA) with addition of 10 µg T4 gene 32 (NEB, Ipswich, MA, USA), according to the manufacturer’s protocol. Double-stranded cDNA was purified using AmpureXP beads (Beckman Coulter, Danvers, MA, USA) and then quantified and quality checked with Bioanalyzer high-sensitivity DNA chips (Agilent, Santa Clara, CA, USA). rRNA depletion was not performed, and rRNA sequences were instead removed computationally using SortMeRNA (51).

Illumina library construction was completed as previously described (53, 54). Libraries were quantified by quantitative PCR on an StepOne Plus system (Applied Biosystems, Foster City, CA) using the KAPA library quantification kit (KAPA Biosystems, Wilmington, MA, USA) according to the manufacturer’s instructions. RMR technology was used to sequence metatranscriptomes and metagenomes from three separate locations in the Kansas native prairie (soils A, B, and C) using MiSeq (300-bp paired ends) and HiSeq (250-bp paired ends) technologies (Illumina, San Diego, CA, USA) for RNA and DNA, respectively.

### Metagenome and metatranscriptome analysis.

The paired-end shotgun reads for both metagenomics and metatranscriptomics analyses were overlapped and quality filtered as outlined in references 52 and 53. The resulting decontaminated (i.e., φX174) reads were then trimmed for quality at <Q_25_ by using the trimmomatic program (54). Short reads generated previously at JGI were 100-bp, paired-end reads and were trimmed to ~80 bp. Rapid mode reads from replicate metagenomes and metatranscriptomes from Kansas soils A, B, and C were trimmed to ~225 bp. Both SR and RMR, were assembled using MEGAHIT (25) with mixed k-mers (21 to 121 k).

The contigs were annotated using MetaPathways2 as previously described (52, 53). Metatranscriptomes and metagenomes were mapped to assemblies and genome bins using Bowtie2 (31). The gene counts per enzyme (EC number) and transcript reads per EC number were mapped onto the Moleculo annotation metabolic pathways in the BioCyc framework (55). Full-length 16S rRNA gene extraction from Moleculo and RMR contig data was completed within Metapathways2 by using LAST (E value, <1E^−7^) annotations with the Greengenes database. 16S rRNA genes from Moleculo-only contigs and RMR contigs were chimera checked with the VSEARCH implementation of UCHIME *de novo*, followed by UCHIME ref against the Greengenes database. Four sequences were initially flagged as potential chimeras, but upon manual investigation we found that low-quality bases in the reference database were the source of mismatches that spuriously inflated UCHIME scores and flagged these reads as chimeric. The data analysis workflow is outlined in Fig. S5 in the supplemental material.

10.1128/mSystems.00045-16.5 Figure S5  Bioinfomatic analysis workflow (further outlined in references 52 and 53). Raw paired-end Illumina reads (.fastq format) have been extended for overlaps by using FLASH (https://ccb.jhu.edu/software/FLASH), after which φX174 is removed using Bowtie2 (BT2), the reads are trimmed with trimmomatic, and then quality control is performed with FastQC (http://www.bioinformatics.babraham.ac.uk/projects/fastqc/). Rapid mode reads (250 bp, paired end) and short reads (100 bp, paired end) are assembled with MegaHit, and the Moleculo data are assembled using the Illumina BaseSpace TruSeq hybrid assembler, which is a string graph-based assembler. For the hybrid assembly, 4-kbp contigs are extracted from the various assemblies (rapid mode reads [RMR], short reads [SR], and Moleculo only) and then are further assembled with the CAP3 overlap consensus assembler. Then, the resulting contigs are further merged using the program minimus2. The resulting contigs at each assembly step are quality controlled and checked with custom python scripts (CPS), which provide assembly size, the number of contigs, contig length distribution, and *N*_50_ values. The quality-controlled contigs reads are mapped back using BT2, annotation is completed with Metapathways2, and data are binned using MaxBin. R and CGviewer are used for visualization. Download Figure S5, PDF file, 0.1 MB. Copyright © 2016 White et al. 2016 White et al. This content is distributed under the terms of the Creative Commons Attribution 4.0 International license.

### Moleculo long-hybrid-read subassembly library preparation and analysis.

Our Moleculo library preparation and long-hybrid-read subassembly using Illumina standard protocols are outlined in Fig. S1 and S5 in the supplemental material and also in the original description of Moleculo (20). DNA from the nine replicate extractions from the A, B, and C soil samples were pooled into a single long-hybrid library prep, following the manufacturer’s protocol (Illumina). The resulting data were assembled on BaseSpace by using the Illumina TruSeq long-read assembly app v1.0.

### Combined assembly merge, genome binning, and genome bin annotation.

After SR (~80-bp) and RMR (~225-bp) reads were assembled with MegaHit into contigs, they were pooled with the Moleculo subassembled reads and further assembled using CAP3 (26). Pooled contigs from the various formats were selected for >4-kbp contigs and then assembled with CAP3 and merged using minimus2 (21–24, 27, 28). Contigs of >1 kbp were binned using MaxBin (32–34) with 107 HMM marker genes and then parsed using the R program. Genome bins were then quality checked with mapping reads using Bowtie2. Annotation and metabolic reconstruction of contigs and genomic bins were completed using Metapathways2 (30), the FOAM database (56), and hmmer3.1 (57) to obtain a list of KEGG ontology numbers. The taxonomic and phylogenetic identifications of the genome bins were obtained using the JspeciesWS Tetra Correlation Search (TCS) (58), and then cross-referenced annotations were obtained by using Metapathways2 and FOAM.

MaxBin binning of short reads from JGI resulted in a novel ~99% complete genome based on 107 HMM markers; this genome was named “*Candidatus* Pseudomonas sp. strain JKJ-1.” Short and Moleculo subassembled contigs were mapped using Bowtie2 and then used for error correction. Genome circulation and closure of “*Candidatus* Pseudomonas sp. strain JKJ-1” was completed by using minimus2 and then finished with the EMBOSS union script followed by manual checking (59). The genome was plotted using the CGviewer server with default parameters (60). The whole-genome-based alignment tree was constructed in the Department of Energy Systems Biology Knowledge Base (KBase; http://kbase.us), using COG domains and FastTree2 (50). Annotation was conducted on the RAST Web server with RAST gene calling based on FIGfam, release 70, and full metabolic reconstruction was performed using ModelSEED (61).

### Nucleotide sequence accession numbers.

The RMR and Moleculo-only 16S rRNA sequences have been deposited in GenBank under accession numbers KX239231 to KX239311 and KX239037 to KX239230. All other data have been deposited in the SRA under our manuscript title, with accession numbers listed in Table S2 of the supplemental material.

10.1128/mSystems.00045-16.7 Table S2  NCBI accession numbers and metadata associated with assembled contigs, unassembled reads, and 16S PCR amplicons. Download Table S2, XLSX file, 0.005 MB. Copyright © 2016 White et al. 2016 White et al. This content is distributed under the terms of the Creative Commons Attribution 4.0 International license.
