# Supplementary figures and images for: Moleculo Long-Read Sequencing Facilitates Assembly and Genomic Binning from Complex Soil Metagenomes
Source: mSystems. 2016 Jun 28;1(3):e00045-16. doi: 10.1128/mSystems.00045-16 (PMC5069762; doi:10.1128/mSystems.00045-16)

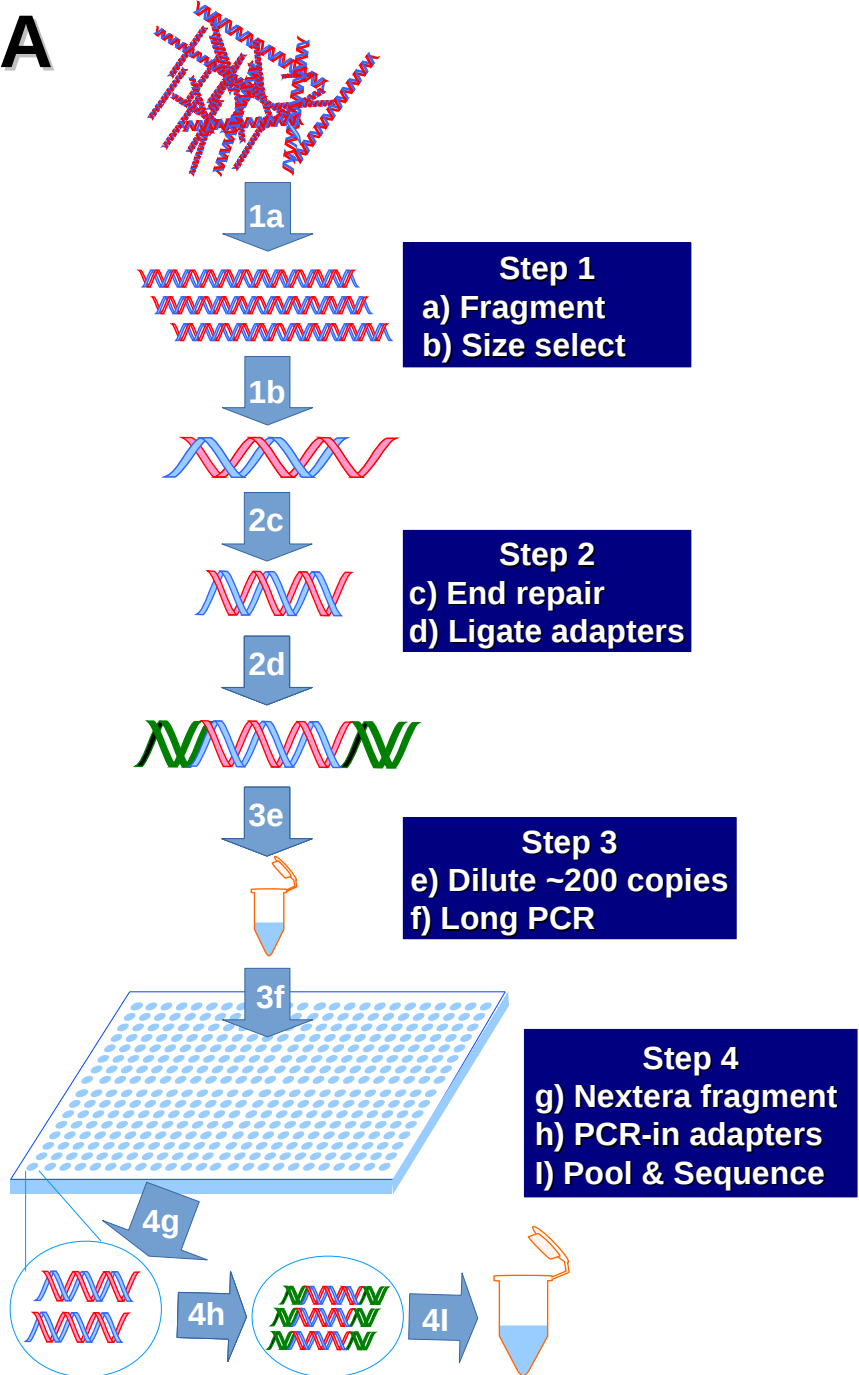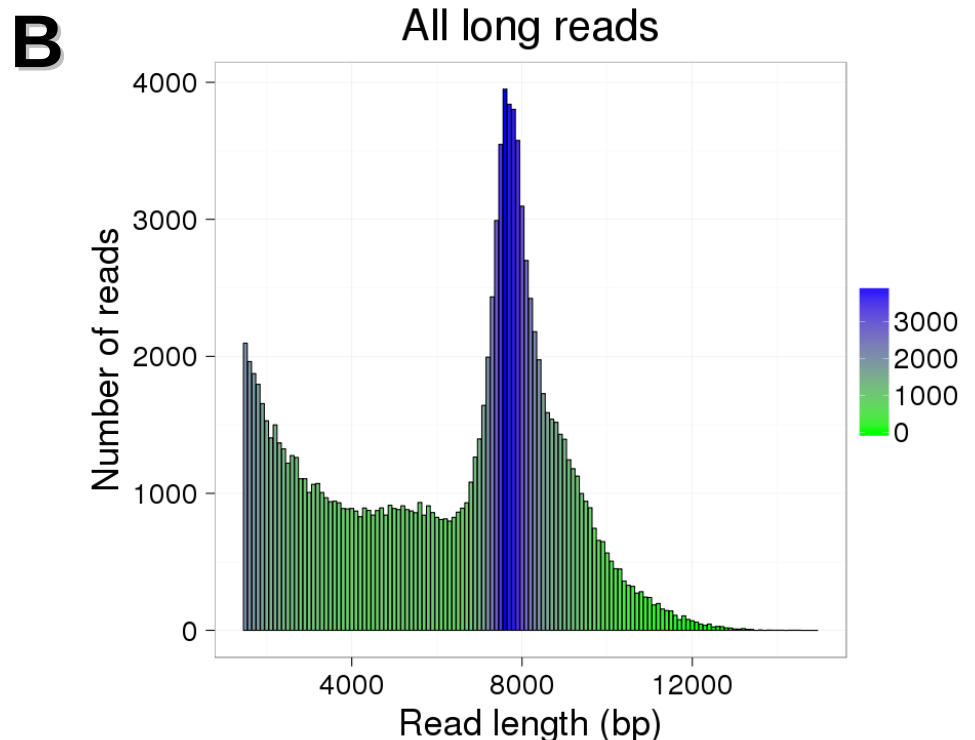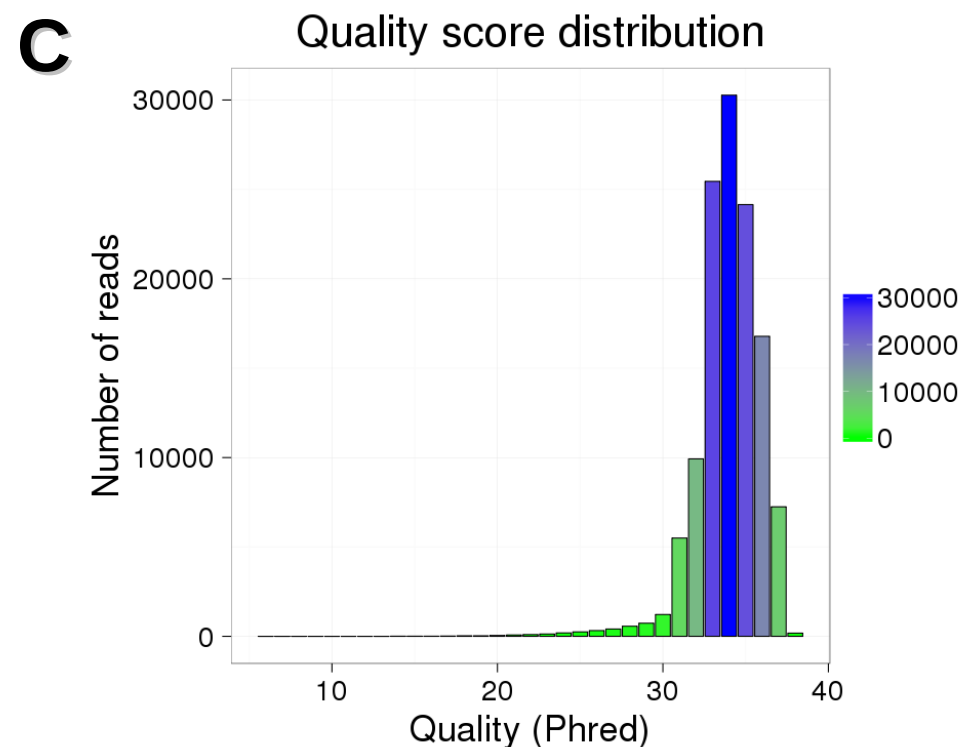

Figure S1

Supplement: Figure S1 [file sys003162031sf1.pdf]

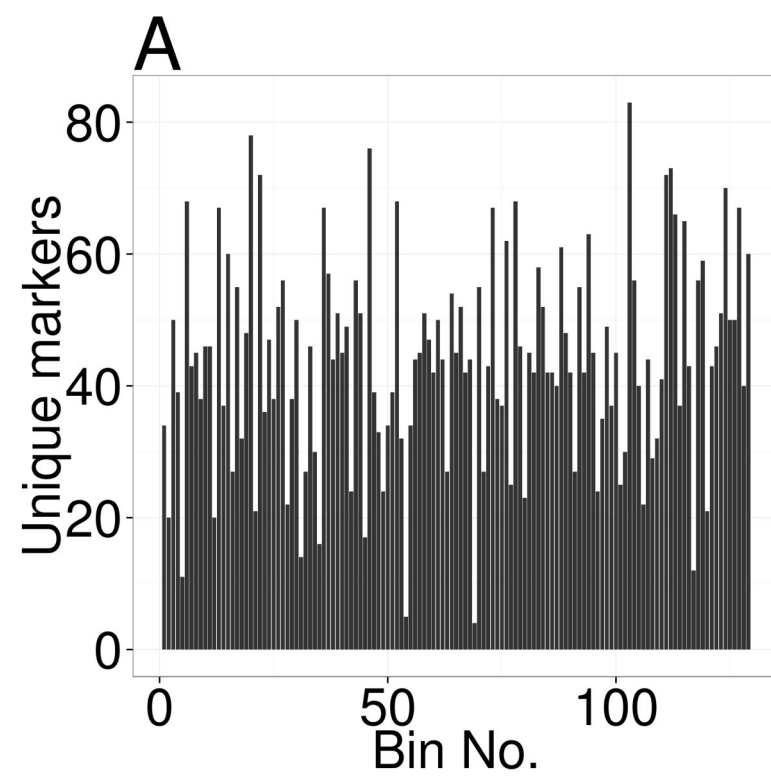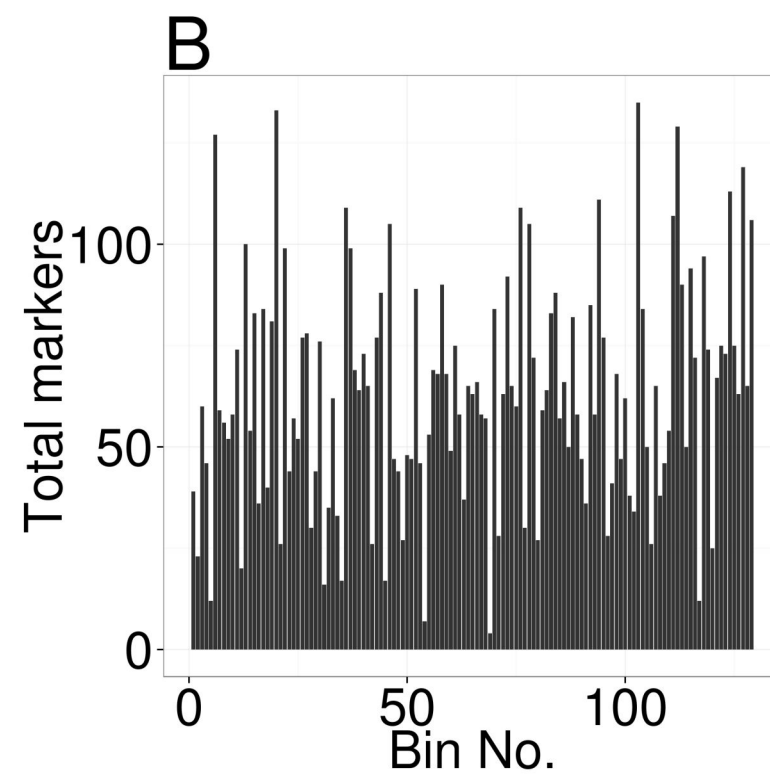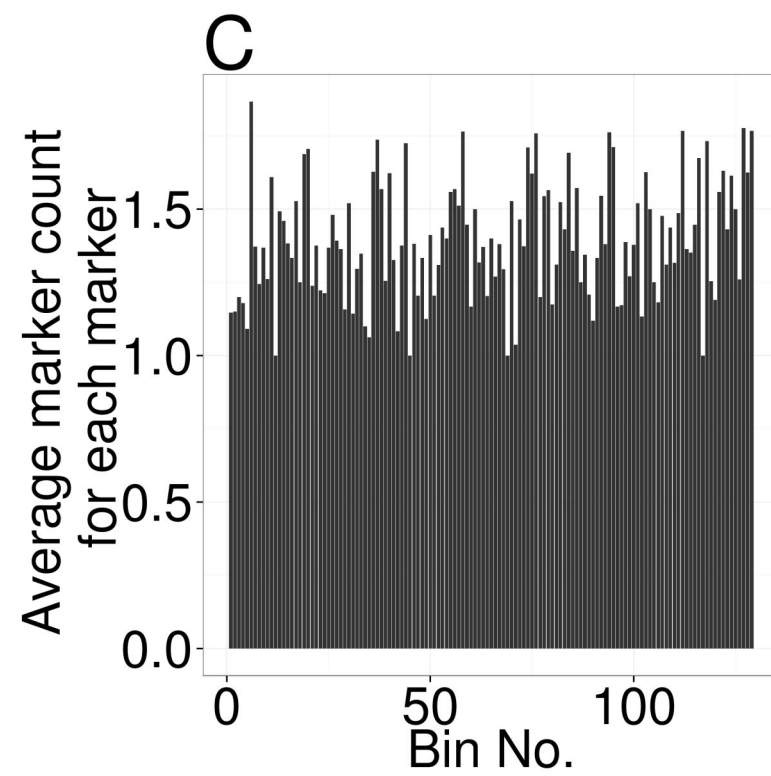

Figure S2

Supplement: Figure S2 [file sys003162031sf2.pdf]

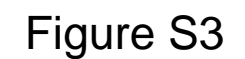

Figure S3

Supplement: Figure S3 [file sys003162031sf3.pdf]

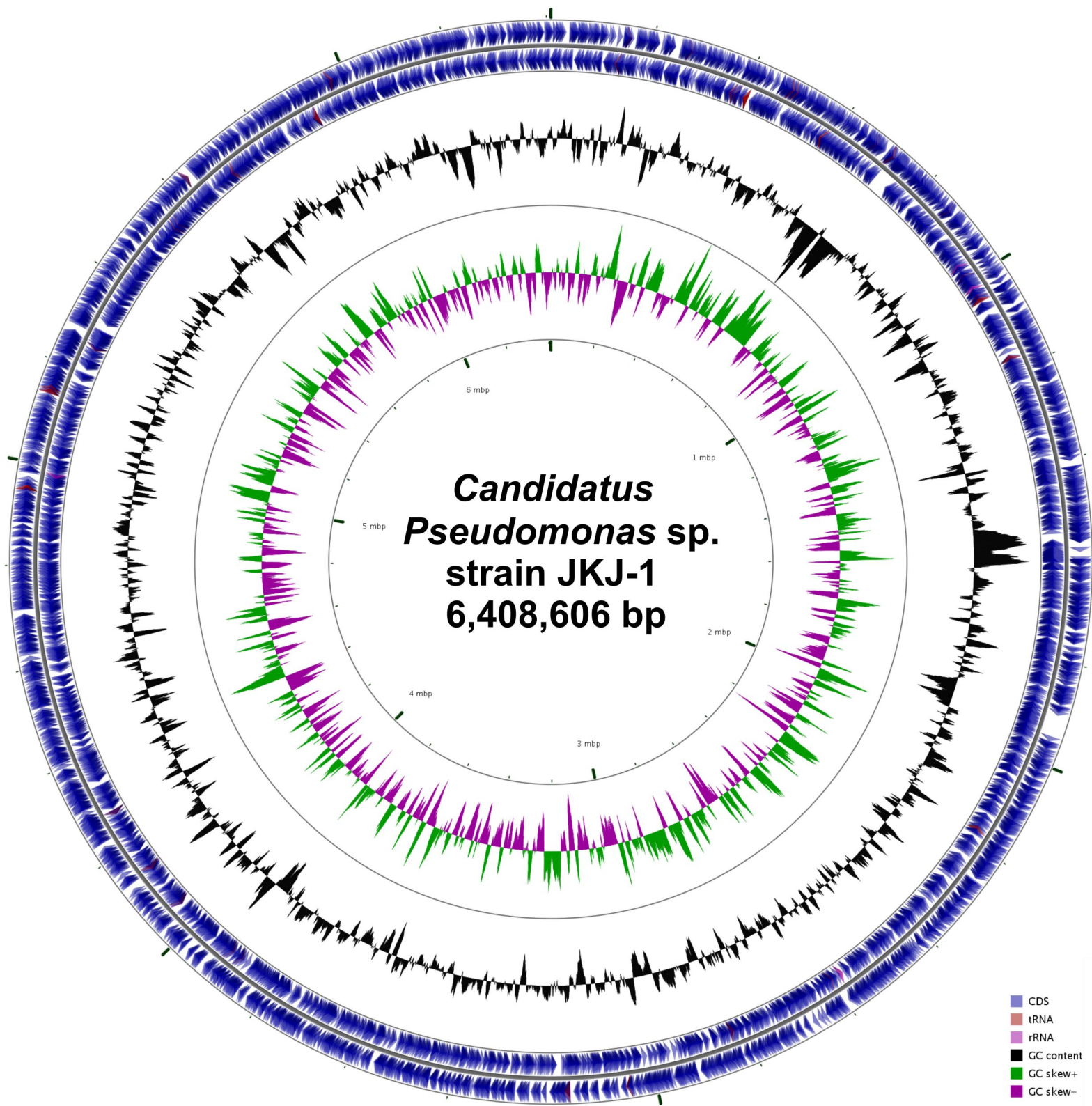

Figure S4

Supplement: Figure S4 [file sys003162031sf4.pdf]

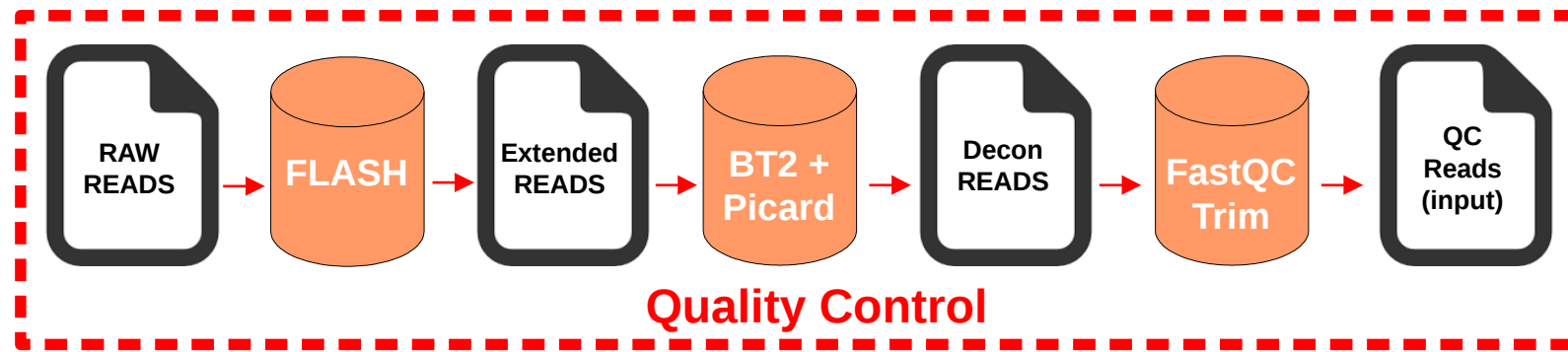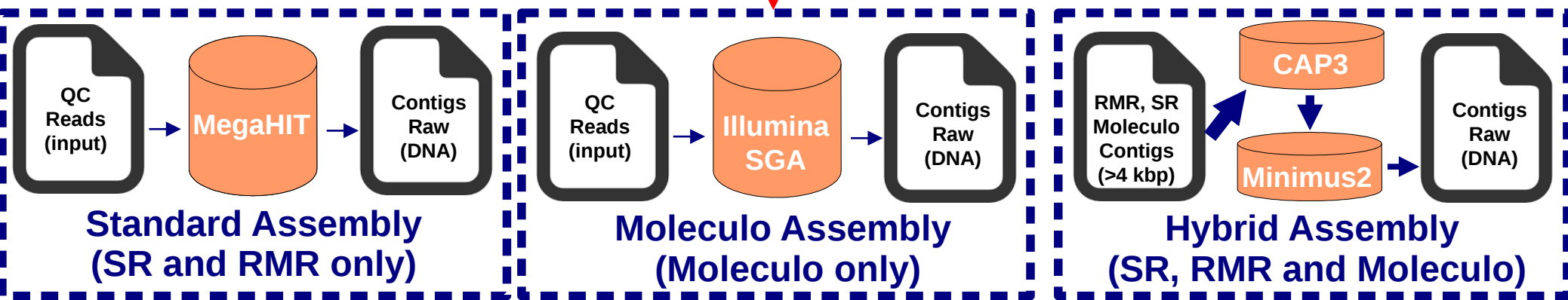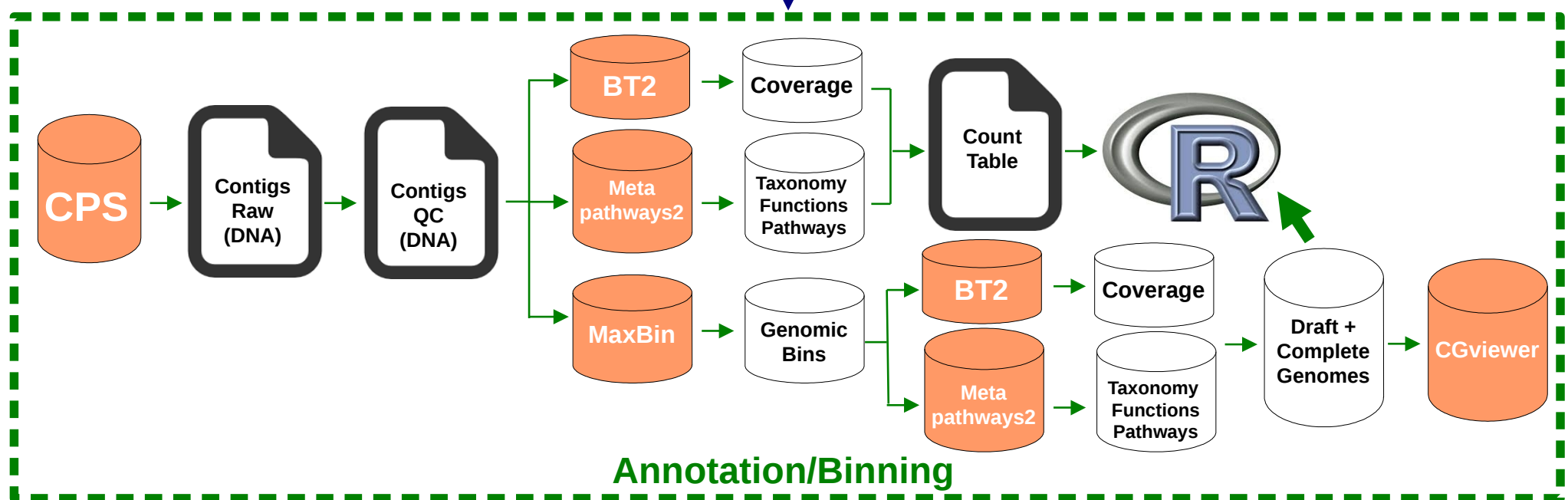

Figure S5

Supplement: Figure S5 [file sys003162031sf5.pdf]
